# Treatment of White Swelling

**Published:** 1846-11

**Authors:** 


*Treatment of White Swelling.*—We have mentioned, on some former occasion, that the Legislature of Massachusetts recognizes no higher claim for services on the part of the most eminent surgeon in the State, than of a wood-sawyer who sets up for a medical and surgical adviser. The following case illustrates this state of things in courts of law. Those who happen to reside where the profession is actually appreciated for the services it renders to the community, ought to be thankful for their position. Again, in this trial, it will be seen that when doctors disagree, the question will no longer be propounded, who shall decide? A jury of twelve men, good and true, soon bring all disputed matters to a focus. Dr. Strong stood valiantly for his opinion, but Dr. Warren's reputation seems to have been heaviest in the balance.

*Court of Common Pleas. Simon C. Hewitt vs. Bedford Lincoln.*—This was an action to recover fifty dollars, for professional services rendered by the plaintiff to the defendant's son. The rendering of the services was not disputed; but the defendent contended that his son was unskilfully treated by the plaintiff, and injured by his practice. Drs. Warren and Strong were called by the defendant to testify that, in their judgment, the treatment of the patient was not judicious. Dr. Warren, however, said that there was a difference of opinion among medical men as to the best method of treating such a case, which was that of a white swelling, and that physicians of eminence and skill adopted the course pursued by the plaintiff. Dr. Strong did not agree with Dr. Warren, that this was a case of white swelling, but pronounced it to be a *synovial inflammation*, and the plaintiff's treatment unsuited to the particular stage of the complaint when the patient was under his care.

The plaintiff's counsel concluded that the opinion of the witnesses, as to the mode of treatment adopted in this case, was a matter of little consequence; that the plaintiff had a system of practice peculiarly his own, in which he had had great success; and that it was a system entirely different from that practiced by the defendant's witnesses. It was not to be expected that practitioners of different schools should approve of each other's practice. They all stood equal before the law, and here no school could set up its standard as the one to which others were bound to conform. If the plaintiff treated the patient properly, according to the principles of his own system, he was entitled to recover, however much that system differed from that of others.

The court instructed the jury that this doctrine was true, so far as the practice of medicine was concerned; but that in surgery a different rule obtained; that there could be but one right method here, and that there must be some standard; that if the jury were satisfied from the evidence that the patient was skilfully treated, they would find for the plaintiff, otherwise they would find for the defendant.

The jury returned a verdict for the plaintiff, for $15.—*Boston Med. and Surg. Jour.*

